# Calprotectin as a smoldering activity detection tool and renal prognosis biomarker in ANCA associated vasculitis

**DOI:** 10.1371/journal.pone.0205982

**Published:** 2018-10-22

**Authors:** Laura Martinez Valenzuela, Juliana Draibe, Maria Quero Ramos, Xavier Fulladosa Oliveras, Edoardo Melilli, Josep Maria Cruzado Garrit, Juan Torras Ambrós

**Affiliations:** 1 Nephrology Unit, Bellvitge University Hospital, Hospitalet de Llobregat, Spain; 2 IDIBELL Biomedical research institute, Hospitalet de Llobregat, Spain; 3 Clinical Science Department, Medicine College, Barcelona University, Hospitalet de Llobregat, Spain; International University of Health and Welfare, School of Medicine, JAPAN

## Abstract

**Background:**

Calprotectin is produced by neutrophils and macrophages, and released during the acute phase of the ANCA vasculitis. The aim of our study was to determine if serum and urine calprotectin are disease activity and prognosis biomarkers in ANCA vasculitis patients during remission.

**Methods:**

Forty-two ANCA vasculitis patients were included. Twenty-seven patients were in remission phase under immunosuppressive therapy, and 15 patients were in the acute phase. Four healthy controls were included. We determined calprotectin in serum and urine samples at the time of the inclusion. We recorded the incidence of relapse and the evolution of GFR, proteinuria, hematuria, and C reactive protein and ANCA titer during 24 months of follow-up.

**Results:**

In remission phase, serum calprotectin was higher than in healthy controls but lower compared to acute patients (p = 0.05). Serum calprotectin at inclusion was higher in patients who increased proteinuria during follow-up (p = 0.04), with hematuria (p = 0.08), and with non-decreasing ANCA titer (p = 0.0019). Serum calprotectin at inclusion in stable patients who subsequently decreased GFR during follow-up was higher compared with those with a stable or improving GFR (p = 0.03). Urine calprotectin was lower in patients with *sclerotic* histology in remission (p = 0.03) and acute phase (p = 0.12) compared to the rest of histologies.

**Conclusions:**

Worsening of renal function, hematuria, rising proteinuria and non-decreasing ANCA correlated with higher levels of serum calprotectin at recruitment. Low urine calprotectin was found in patients with *sclerotic* histology. Calprotectin during remission in ANCA vasculitis may be useful to identify subclinical inflammation and worse renal prognosis patients.

## Introduction

Anti-neutrophil cytoplasmic antibodies (ANCA) associated vasculitis (formerly AAV) is a life-threatening disease that usually affects kidney, with a wide spectrum of severity that includes rapidly progressive renal failure.

ANCA are crucial for the pathogenesis. These antibodies are directed against proteinase 3 (PR3) or myeloperoxidase (MPO), enzymes located inside cytoplasmic granules of neutrophils and macrophages. Because of unknown factors (genetical, environmental or infectious) cells are primed and expose those enzymes on their surface, with subsequent anomalous formation of pathogenic antibodies. Binding of antibody causes cell activation and degranulation which damages endothelium, the hallmark of the disease[1]. Glomerulonephritis in AAV is characterized by infiltration of the glomeruli by neutrophils, macrophages and T cells, and is not mediated by immunocomplexes.

Management of the disease is based on immunosuppressive (IS) drugs, but the duration of maintenance treatment has not yet been defined in randomized controlled trials. Clinical practice guidelines recommend at least 24 months with a low grade of evidence[2].Sanders et al found no differences in relapse-free survival under azathioprine treatment comparing one or four years schedules of treatment[3]. On the other hand, the REMAIN study showed lower relapse incidence among patients treated with azathioprine/prednisone for 48 months compared to those treated for 24 months, suggesting an additional contribution to the relapse-free survival by corticosteroids[4]. Moreover, a Japanese study found a higher relapse incidence in remission patients treated with 2.5mg of prednisone/day compared with patients treated with 5mg/day[5]. Further studies are needed to define the dosage and especially the duration of the maintenance treatment, since the main drawback is the identification of the activity of the disease.

In this line, research in AAV biomarkers is crucial to identify patients at higher risk of poorer outcomes, to modulate IS treatment and to balance risk of relapse and treatment adverse effects. Main risk factors accepted for the appearance of future relapse are history of prior relapse and persistence of hematuria after apparent clinical remission. Inflammatory markers as C-reactive protein (CRP) are broadly used in clinical practice to assess disease activity, but they are highly unspecific. Unfortunately, the value of the ANCA titer to predict disease activity is controversial. Kemna et al showed a correlation between ANCA titer rise and disease relapse in a subgroup of 104 AAV patients with renal involvement[6]. On the other hand, a meta-analysis showed that increasing ANCA titer during remission was barely predictive of relapse[7]. Lionaki et al proved that ANCA PR3 specificity predicts relapse in AAV patients with kidney involvement, conferring almost twice relapse risk compared to ANCA MPO vasculitis[8].Several other biomarkers are being evaluated, as antibodies against lysosome-associated membrane protein-2 or moesin, or diverse lymphocyte subsets, with non-robust results until present[9].

Calprotectin (S100A8/S100A9, MRP8/14) is secreted by activated neutrophils, monocytes and early differentiated macrophages[10]that acts as a damage-associated molecular pattern and Toll-like receptor 4 activator[11]. It is released after cell contact with certain extracellular matrix proteins. Calprotectin binds endothelial cells and amplifies endothelial damage and inflammation, by increasing IL8, ICAM1 and leukocyte recruitment[12]. Calprotectin release has been identified by immunohistochemistry in glomerular infiltrating macrophages in active lesions on kidney biopsies of AAV, but not in sclerotic areas[12], confirming its role as inflammatory mediator in this disease. Serum calprotectin is elevated in patients in the acute phase of AAV compared to patients in remission, but during remission the normal levels are not reached[12]. As shown in a recent study, an increase in serum calprotectin by month 2 or 6 compared to diagnostic time in rituximab treated patients was an independent risk factor for relapse of the disease in PR3 AAV[13]. Serum calprotectin has shown its utility as a marker of activity and relapse in other autoimmune diseases[14].On the other hand, the usefulness as biomarker of urine calprotectin AAV has not been well evaluated although it has been proven as an indicator of parenchymatous acute renal failure when inflammation is present[15].

So, reliable biomarkers are urgently needed to better identify or predict the AAV relapse. The aim of the present study was to prospectively explore the value of serum and urine calprotectin as biomarkers in AAV patients throughout a stability period during disease remission. To our knowledge, this is the first time that calprotectin is evaluated as a biomarker of relapse, activity, and renal prognosis in AAV patients in prolonged stable remission patients.

## Materials and methods

As study population, we included 42 AAV patients: 15 patients in acute phase of the disease and 27 patients in remission quiescent phase of the disease. Regarding to the acute cohort, 10 out of the 15 patients were treatment-naïve and included at the moment of their diagnosis, and the remaining 5 were on a disease flare. The remission cohort comprised 27 patients diagnosed with AAV with renal involvement not dialysis-dependent, in the remission phase of the disease after successful induction response, which were prospectively and periodically followed at *Bellvitge University Hospital*. All patients were clinically stable at inclusion and were not affected with neoplasms, active infectious disease (including patients with prominent leukocyturia and/or positive urine culture), or any other active autoimmune disease prior to the study that could also elevate calprotectin levels. All patients were under a similar therapeutic scheme, according to the standard clinical practice at our institution. Induction to remission was achieved by means of plasma exchange or intravenous metilprednisolone pulses followed by oral or intravenous cyclophosphamide, and maintenance treatment consisted on similar doses of oral prednisone(2.5–5 mg daily) and mycophenolate mofetil (500mg twice a day) or mycophenolic acid (360mg twice a day) as supported by the latest EULAR/ERA-EDTA guidelines[2]. 4 healthy controls were also included.

In all cases, the primary diagnosis was made according to the presence of typical signs and symptoms, positivity of ANCA antibodies in serum and histological findings[16]. Renal involvement was confirmed by kidney biopsy in all cases. Remission of the disease was defined by the absence of signs and symptoms of vasculitis (Birmingham Vasculitis Activity Score (BVAS) 0[17]), and relapse was defined as the appearance of 1 out of the 24 items of the BVAS indicating threatened function of a vital organ attributable to vasculitis or appearance of at least three other BVAS items. All patients were prospectively controlled at our outpatient’s clinic for a minimum of 24 months by an experienced medical team and had available medical records. The Ethical Committee of Bellvitge University Hospital approved the study protocol. All the engaged patients signed informed consent.

The primary goal was to evaluate calprotectin as disease activity biomarker in AAV during remission. The secondary goal was to evaluate calprotectin as a prognosis marker of the progression of GFR and proteinuria through time in patients in remission as surrogate markers of subclinical activity.

We determined serum and urine calprotectin at the moment of the recruitment (we will refer to this value as *baseline* value), and serum calprotectin at the end of the follow-up period using a commercial ELISA kit(Legend Max Human MRP8/14, Biolegend inc., San Diego, CA) following manufacturer’s instructions. No intermediate sampling was made between these two timepoints.

We collected clinical and analytical data from medical records. We recorded sex, age, time since diagnosis, history of extra-renal manifestations and prior relapses. We assessed disease activity by means of hematuria, proteinuria, ANCA titer and specificity, and CRP. The cut-off value for ANCA titer in our institution is 5 karb.u./L, and we considered a rise in ANCA titer when it increased more than 5karb.u./L during follow-up and a decrease in ANCA titer a reduction higher than 5karb.u./L. The cut of value for CRP is 5ng/L in our institution, and we considered an increase or decrease of any magnitude to our analysis. We evaluated the outcome of kidney function according to serum creatinine measurement and GFR by the CKD-EPI formula. GFR worsening was defined as a decline in GFR higher than 2mL/min/year, as physiologic GFR loss is considered 1mL/min/year. Histology in the diagnosis was analyzed according to the Berden classification[18].

Data was analyzed using GraphPad Prism version 6.00 (GraphPad Software, La Jolla California USA) and IBM SPSS Statistics Version 20.0 (IBM corp., Armonk, NY, USA). D'Agostino & Pearson omnibus normality test was applied to determine whether quantitative variables were or not normally distributed. For comparison of two groups, Student’s T test was used when variables were quantitative and normally distributed and Mann-Whitney U if they were not normally distributed. For comparison of more than two groups, ANOVA test was used for normally distributed variables and Kruskal-Wallis test for non-normally distributed. Chi-square test was used for qualitative variables. Correlations were assessed using Spearman’s or Pearson correlation, depending on the distribution of the variable. Univariate binary logistic regression was used to assess if measured variables had a causal association with the observed outcomes. P values less than 0.05 were considered significant.

## Results

### Baseline and clinical outcome

Baseline characteristics of the remission and the acute cohort are summarized in Table 1.

**Table 1:** Baseline characteristics of the population.

	Remission Cohort (n = 27)	Acute cohort (n = 15)
**Sex (%)**		
**Men**	40.7	33.3
**Women**	59.3	66.7
**Mean age±SD (years)**	66.1±12.5	62.9±14.7
**Median time from diagnosis (Range) (months)**	48(18–119)	Diagnostic patients: 0 Relapse patients: 36
**ANCA specificity (%)**		
**MPO**	74	86.6
**PR3**	26	13.3
**ANCA titer**		
**Persistently positive (%)**	48.14	100
**Negativized during follow-up (%)**	51.86	0
**ANCA titer**	63.94±148.6	119.2±169.2
**Chapel Hill Classification (%)**		
**MPA**	62.9	80
**GPA**	29.6	20
**EGPA**	7.4	0
**Mean serum creatinine±SD (μmol/L)**	165.1±62.4	296.3±319.8
**Mean GFR±SD (mL/min)**	39.6±24.2	33.9±27.1
**Hematuria (%)**	25.9	86,6
**Mean proteinuria±SD (gr/mol)**	42.07±37.5	106.7±96.6
**Mean CRP±SD**	5.7±13.5	47.9±50.1
**Berden Histopathologic Classification (%)**		
**Sclerotic**	18.5	25
**Focal**	22.2	33.3
**Crescentic**	11.1	16.6
**Mixed**	33.3	25
**Previous relapses (%)**	33	0
**Induction treatment**		NE
**Cyclophosphamide (%)**	66.7	
**Plasma exchange (%)**	25.9	
**Lung involvement (%)**	33.3	13.3

SD–standard deviation, ANCA- Anti-neutrophil cytoplasmic antibodies, MPO- myeloperoxidase, PR3- proteinase 3, MPA- microscopic polyangiitis, GPA- granulomatosis with polyangiitis, EGPA eosinophilic granulomatosis with polyangiitis,GFR- glomerular filtration rate, CRP- C reactive protein.NE Non evaluable

The median time since diagnosis to recruitment for the 27 patients included in the remission period was 48 months, ranging from 18 to119, and the median time from remission to inclusion in the study of these patients was 22 months, range 7–118. Mean follow-up was 50.4±8.7 months. 51.9% patients presented ascending CRP during follow-up and 44.4% patients presented rising ANCA titer. 4 out of 27 patients experienced a relapse (14.8%) at a mean time of 11±10.5 months after recruitment.

**Fig 1:**
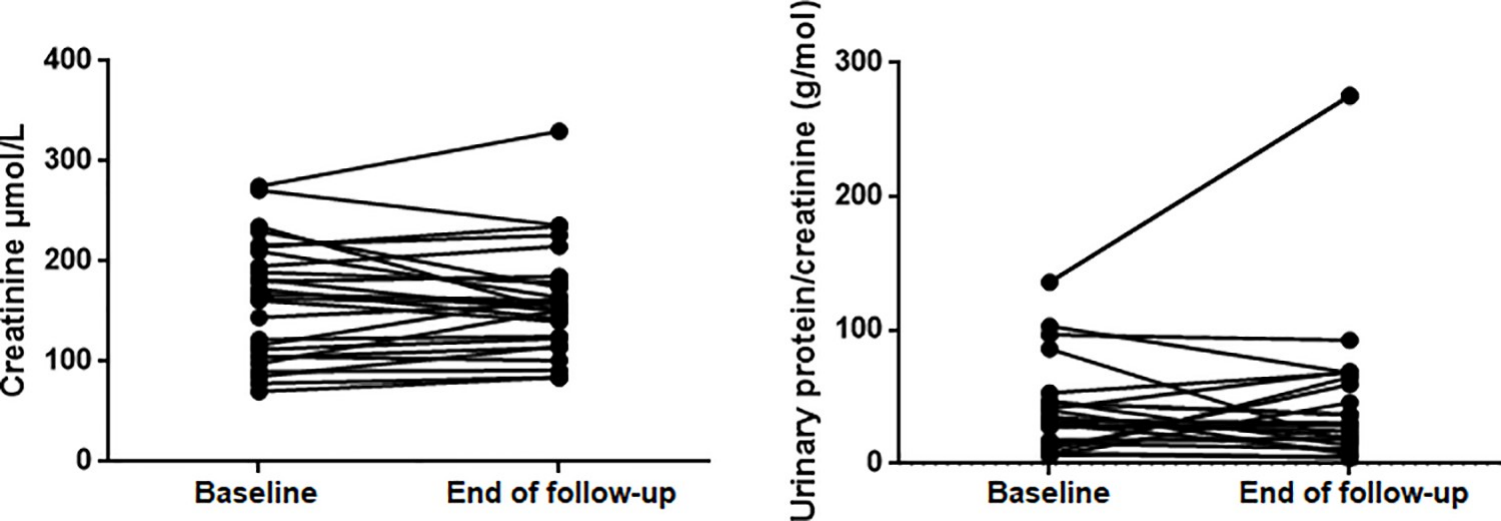
Evolution of serum creatinine and proteinuria in the remission cohort.

Regarding the evolution of the renal function,62.9% of patients in the remission cohort presented a rise of any magnitude in serum creatinine (mean creatinine rise20.1±18.2 **μ**mol/L) and 37.1% patients increased proteinuria during follow-up, although differences between baseline and follow-up values did not reach signification. (Fig 1).14.8% of patients maintained or presented new-onset hematuria. Only 2 out of 27 patients required initiation of renal replacement therapy. (See Table 2).

**Table 2:** Evolution of analytical parameters.

	Baseline	End of follow-up	P value
**Serum Creatinine (μmol/L)**	165.1±62.4	159.8 ±55.3	0.86
**Proteinuria ratio (g/mol)**	42.1±37.5	53.2±71.1	0.36
**CRP (mg/L)**	5.7±13.5	6.1±10.1	0.89
**ANCA titer (karb.u./L)**	63.9±148.0	23.7±29.3	0.11
**Hematuria (%)**	60.9	14.8	0.9

Baseline and final serum creatinine, proteinuria, CRP and ANCA titer in the overall cohort of stable patients in remission. Comparison of means did not show significant differences. CRP- C reactive protein. ANCA–anti-neutrophil cytoplasmic antibodies.

### Serum calprotectin in the remission cohort

**Fig 2:**
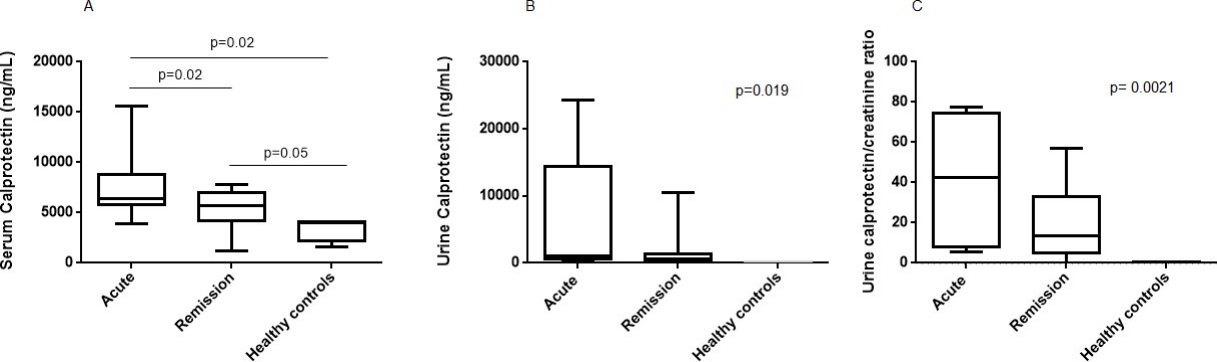
Mean serum, urinary calprotectin and urinary calprotectin indexed to urinary creatinine of the cohort of acute AAV patients, remission patients and healthy controls.

Mean baseline serum calprotectin was 5403±1971ng/mL, and it significantly decreased to 3870±1950ng/mL at the end of the follow-up period (p = 0.02). As expected, serum calprotectin at baseline in our cohort of AAV remission patients was significantly higher thanin healthy controls (5403±1971ng/mL vs. 3401±1212ng/mL respectively, p = 0.05). On the opposite, serum calprotectin was lower in our cohort of remission patients compared to thosein the acute phase of the disease (5403±1971ng /mL vs. 7343±2995 ng/mL respectively, p = 0.02). (Fig 2).

Serum calprotectin in remission patients was independent from time since diagnosis to the recruitment(R^2^ 0.05, p = 0.27), age (R^2^ 0.025, p = 0.46),induction treatment (p = 0.84 for plasma exchange and p = 0.96 for cyclophosphamide) and among categories in Berden Classification (p = 0.65). Neither association between serum calprotectin and GFR (R^2^ 0.009, p = .064) or history of lung involvement of the disease (p = 0.29) was found.

Summarizing, serum calprotectinacts as a marker of disease activity and is independent of variables as treatment, GFR or extra-renal involvement.

### Serum calprotectin as a disease activity biomarker

Mean serum calprotectin was not different in remission patients that relapsed during the follow-up and patients who did not (5057±1072ng/mL vs. 5280±400ng/mL, p-value = 0.86).

On the other hand, baseline serum calprotectin was higher in patients with non-decreasing ANCA titer during the follow-up period compared to those patients who decreased ANCA titer (5816±337.1 vs. 2768±1044 ng/mL, p value = 0.0019). Serum calprotectin did not differ depending on ANCA specificity (p = 0.21) or history of previous relapse (p = 0.73).

**Fig 3:**
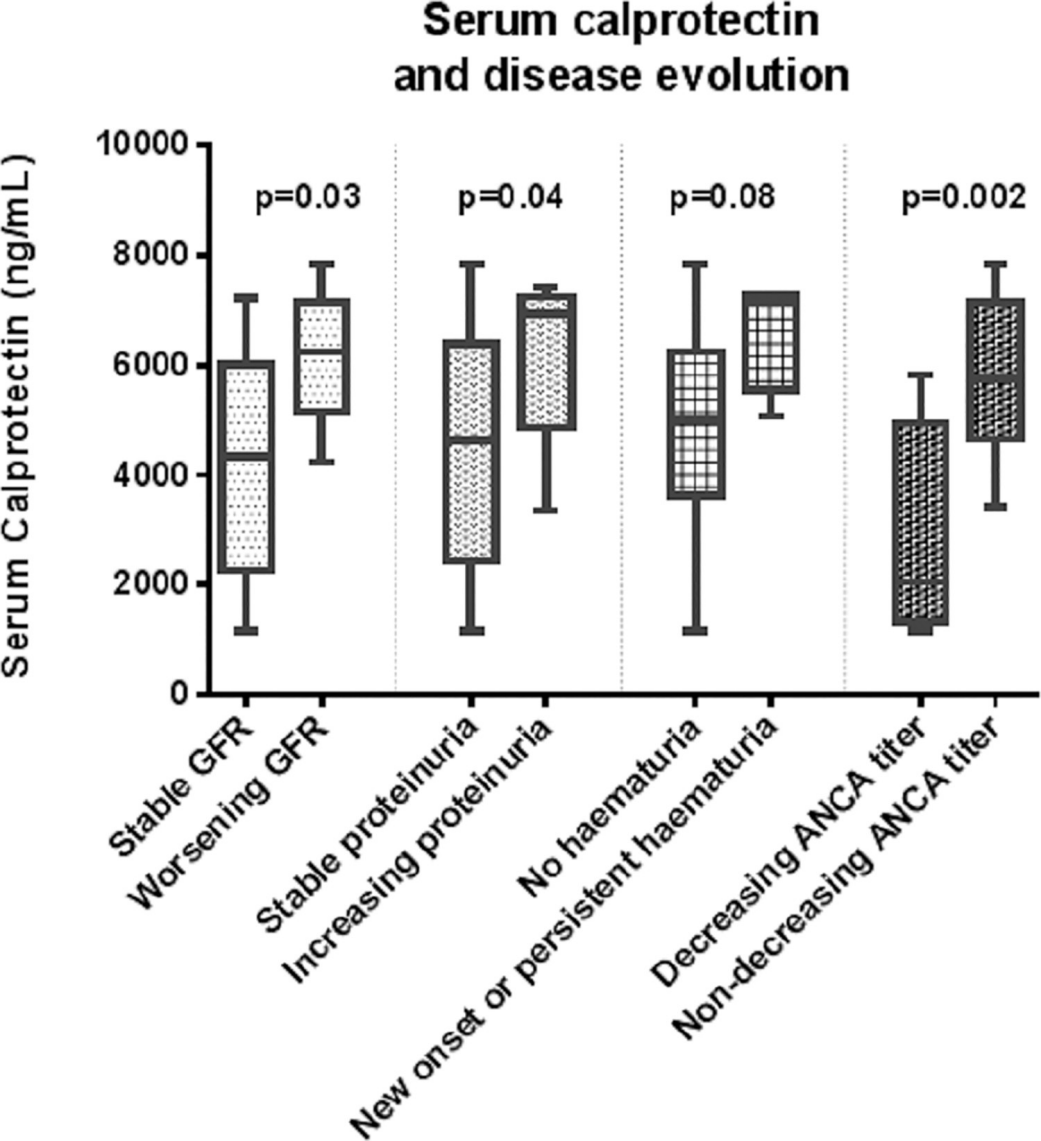
Serum calprotectin and evolution of the different variables recorded. GFR–Glomerular filtration rate. ANCA- Anti-neutrophil cytoplasmic antibodies.

Additionally, patients with hematuria at the end of the follow-up period (this is patients with de novo or maintained hematuria) exhibited a tendency to higher baseline serum calprotectin compared with the rest of the cohort (6652±532.6 ng/mL and 4808±479.4 respectively, p = 0.08). In relation to proteinuria, patients with rising proteinuria during the follow-up had a mean serum calprotectin of 6017±465.2 ng/mL, meanwhile it was 4525±521.6ng/mL in patients with stable or decreasing proteinuria (p = 0.04). Of interest, baseline proteinuria was not different between patients with rising and non-rising proteinuria(p = 0.82) (Fig 3). Finally, CRP did not correlate with serum calprotectin (R square 0.0087 p = 0.69).

Summarizing, serum calprotectin failed to predict relapse, but was associated to ANCA rise, proteinuria increase and the presence of hematuria at the end of the follow-up.

### Serum calprotectin as a renal function outcome biomarker

**Fig 4:**
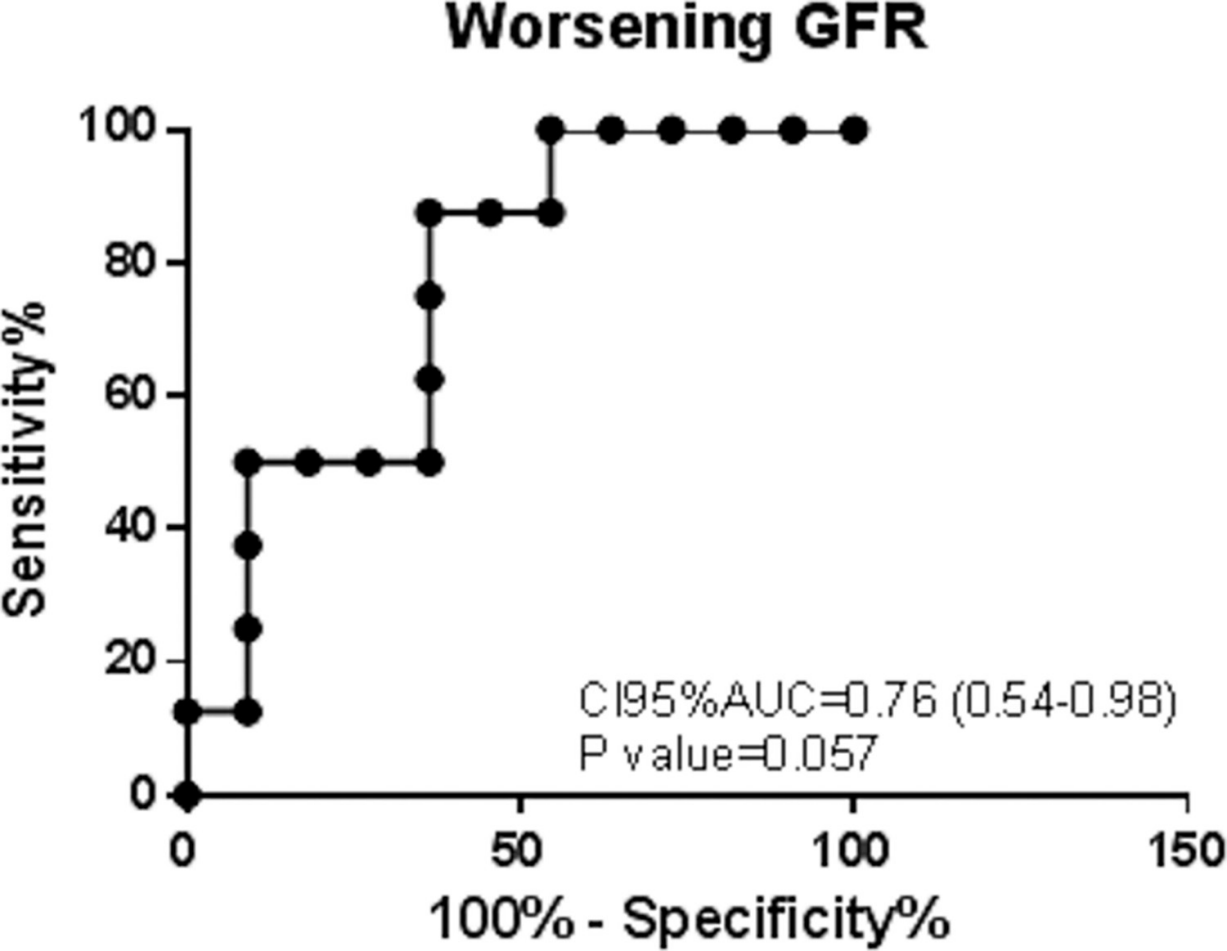
ReceiverOperatorCurve for the levels of calprotectin as a biomarker of worsening GFR. AUC- area under the curve. P–p value.

Patients with worsening renal function during the follow-up had a mean serum calprotectin of 6154±445.3ng/mL, compared to a mean serum calprotectin of 4224±603.6ng/mL in patients with stable GFR (p = 0.03). We plotted a ROC curve to assess the value of serum calprotectin as a predictor for worsening renal function, and we obtained an AUC = 0.76 p value = 0.057 (Fig 4). By univariate binary logistic regression, serum calprotectin level was associated with poorer renal outcome meanwhile age, time since diagnosis, GFR, proteinuria and ANCA titer did not (Table 3).

**Table 3:** Univariate analysis.

	Worsening GFR	Rising proteinuria	Non-decreasing ANCA	New onset/persistent hematuria
	OR(CI 95%), P value	OR(CI 95%), P value	OR(CI 95%), P value	OR(CI 95%), P value
**Age**	0.980(0.916–1.047), 0.543	1.014(0.951–1.082), 0.667	0.992(0.907–1.084), 0.857	1.098(0.958–1.259), 0.178
**Time since diagnosis**	0.996(0.984–1.008), 0.5	0.999(0.988–1.01), 0.821	1.008(0.995–1.021), 0.229	1(0.986–1.014), 0.999
**Proteinuria**	1.006(0.985–1.028), 0.583	1.003(0.982–1.024), 0.810	0.998(0.968–1.028), 0.879	1.010(0.984–1.037), 0.447
**Baseline GFR**	0.977(0.937–1.018), 0.262	0.997(0.964–1.031), 0.856	1.009(0.969–1.051), 0.658	1.004(0.998–1.010), 0.197
**Baseline s-Creat**	1.011(0.996–1.026), 0.149	1.006(0.993–1.006), 0.38	0.996(0.979–1.014), 0.667	0.988(0.968–1.008), 0.236
**ANCA titer**	1.002(0.996–1.008), 0.5	0.997(0.990–1.005), 0.458	1.003(0.997–1.009), 0.271	1.010(0960–1.062), 0.704
**Serum Calprotectin**	1.001(1–1.001), 0.025*	1(1–1.001), **0.104**	0.999(0.998–1) 0.028*	1(1–1.001), 0.313

Table 3 shows univariate analysis for the outcomes registered at the end of the follow up period, depending on the recorded variables. GFR- Glomerular filtration rate; s-Creat–serum creatinine; ANCA–anti-neutrophil cytoplasmic antibodies; OR–odds ratio.

### Urine calprotectin as a disease activity and renal function evolution biomarker

Mean urine calprotectin was 6923±9311 ng/mL in patients in the acute phase, 2893±1766 in remission patients and 68.6±43.9 ng/mL in healthy controls (p = 0.019) (Fig 2). Urine calprotectin indexed to urine creatinine was also higher in acute patients compared to remission patients and healthy controls. Urine calprotectin did not correlate with serum calprotectin or GFR in the remission or acute cohort(R^2^ 0.017 p value = 0.54 and r = 0.04, p value = 0.96, respectively).(See Fig 2)

Urine calprotectin in remission patients showed no differences between patients who relapsed during follow-up and those who did not (p = 0.7), ANCA-MPO and ANCA-PR3 patients (p = 0.5) or ANCA positive and negative patients (p = 0.8). We did not find differences between patients with decreasing ANCA titer compared to patients with non-decreasing ANCA titer (p = 0.64), patients with history of previous relapse (p = 0.2), or patients with persisting or new-onset hematuria or rising proteinuria (p = 0.8 and p = 0.93 respectively). Urine calprotectin showed no differences depending on the evolution of GFR (data not shown).

**Fig 5:**
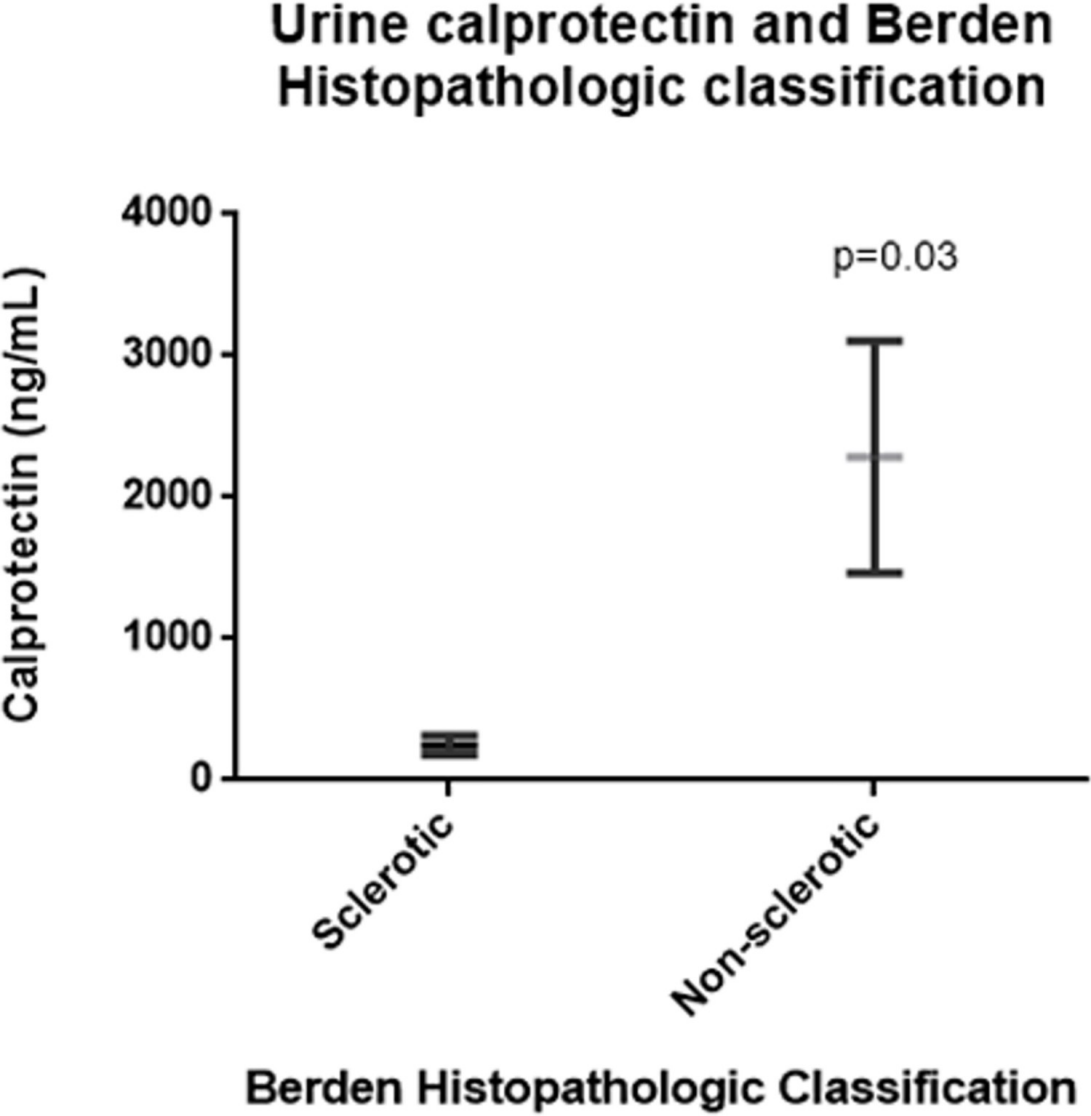
Mean urinary calprotectin in remission patients, according to groups defined by Berden Histopathologic Classification at diagnosis.

Interestingly, urine calprotectin during remission in patients classified as *sclerotic* in the Berden Classification at the time of diagnostic was lower compared to the rest of histologies (248.2±66.9ng/mL vs. 2285±821.2ng/mL; p value 0.03). Moreover, at the cohort of patients in acute phase of the disease, only 25% patients were classified as *sclerotic* and, in this case, we found a bigger difference of urine calprotectin between *sclerotic* and other categories(sclerotic 309.4±767.1 ng/mL vs. others 9991±8384 ng/mL; p value = 0.12).(Fig 5). We did not find differences when urine calprotectin/creatinine ratio was compared among *sclerotic* and the rest of the histological patterns.

## Discussion

Neutrophils play a key role in the vascular endothelial damage in AAV. ANCA are directed against auto-antigens from neutrophils, causing degranulation and release of cytokinesand tissue-damaging molecules[19] which cause endothelial injury. Originating from neutrophils, calprotectin has shown promising results as a biomarker in some autoimmune diseases[20]. A previous study demonstrated a role for calprotectin in AAV pathophysiology as authors evidenced higher serum calprotectin levels in AAV patients, and glomerular infiltration by calprotectin-releasing neutrophils and macrophages[12]. Failure to suppress serum calprotectin was predictive for disease relapse in a cohort of PR3-AAV acute patients treated with rituximab[13]. The goal of our study was to determine the utility of serum calprotectin to detect subclinical activity in a cohort of patients apparently in stable remission of the disease. In addition, this is the first time that the relationship between serum calprotectin and the evolution of GFR, proteinuria and ANCA titer is described. Regarding to urine calprotectin, no study has previously examined urine calprotectin in AAV patients, what also constitutes a novelty of our research.

Here we present a population of AAV patients who were stable in remission after successful induction therapy, who were prospective and exhaustively followed for a long substantial time. The stable remission is reflected by a low rate of relapse and the absence of variations in all evaluated analytical variables.

First, we confirmed previous data regarding the higher serum calprotectin levels in acute AAV patients compared to healthy patients. Interestingly, our study remission population showed serum calprotectin levels higher than healthy controls suggesting that there may exist a smoldering activity of the disease. In addition, we evaluated the evolution of the ANCA titer, proteinuria and hematuria as standard parameters of disease activity in clinical practice. As seen in the results, higher serum calprotectin levels were related to non-decreasing ANCA titer, new-onset/persistent hematuria and rising proteinuria. This confirms that high calprotectin levels may be associated to a more inflammatory profile during disease evolution according to classical parameters. We can assume that the lack of correlation with CRP is due to the high unspecificity of this parameter, and also a lower sensitivity compared to calprotectin in the detection of subclinical activity, as it has been described in rheumathoid arthritis[21].

Unfortunately, we could not find higher calprotectin levels in patients who relapsed during follow-up, probably because the low number of events we registered. Previous studies with acute patients found that the evolution of the biomarker after the diagnosis of the disease was predictive for relapse, but interestingly they neither could establish a serum calprotectin cutoff value as predictor[13]. We analyzed the two-year evolution of serum calprotectin at our cohort and the forward clinical evolution of these patients to assess a possible association with disease activity and relapse episodes, but we think that we need further follow up to identify an association between the two-year evolution of our biomarker and future relapses.

The data obtained from our cohort of AAV patients in the remission phase of the disease, with higher serum calprotectin levels correlating to higher GFR loss during follow-up, suggests that serum calprotectin may be useful to identify those individuals at a higher risk for a faster GFR deterioration.

Urine calprotectin was lower in remission patients classified as *sclerotic* at the diagnostic kidney biopsy according to Berden classification. Infiltrating neutrophils and macrophages produce calprotectin and excrete the mediatordirectly to the urinary space as was proposed by O’Reilly et al on their research in urinary sCD163 in AAV patients[22]. Pepper et al found higher expression of calprotectin in kidney biopsies with intense inflammatory infiltrate, that lacked in scarred glomeruli[12]. In the setting of other autoimmune diseases with presence of inflammatory infiltrate in the kidney, higher levels of urine calprotectin have been reported in comparison to patients with equal degree of kidney impairment of non-inflammatory cause[15]. In consequence, we assume that the finding of higher urine calprotectin in acute patients compared to remission patients and healthy controls may relate to the presence of higher active inflammatory infiltrate in the acute scenario. This rationale might also apply to the lower urine calprotectin seen in sclerotic patients compared to the remainder. Regarding to histopathologic classification, urine calprotectin indexed to urine creatinine was not lower in sclerotic patients compared to the rest. We think that this is, in part, because of the size of the sample. However, it is important to add that there were not differences in GFR between sclerotic patients and the rest of the patients in both cohorts. Importantly, low urinary calprotectin is associated to sclerotic histology in our study, so we speculate that an eventual reduction in urine calprotectin during clinical follow-up may suggest progressive sclerosis indicating futility of intensive IS therapy.

Our biomarker is independent from the kidney function. We consider that this is essential, given the fact that AAV is a chronic disease that frequently alters GFR, thus consolidating calprotectin as a solid biomarker in AAV.

The size of our study cohort may represent an important limitation of our study, mostly because the relapse of the disease was infrequent. We are now progressing on the prospective inclusion of a higher number of patients to further validate the association between urine and serum calprotectin and relapse, alike as the association between calprotectin and disease activity has been proven in this study. Follow up period should be extended to elucidate if 2-year calprotectin evolution is predictive for future events.

In summary, we were able to demonstrate the association of the serum calprotectin levels with surrogate disease activity markers and evolution of the renal function. Regarding to urine calprotectin, we demonstrated lower levels in sclerotic histology, maintained during the remission phase. All together, we think that the information derived from serum and urine calprotectin quantification is helpful to individualize immunosuppressive maintenance therapy in AAV.

## Supporting information

S1 Table Database. Supplementary table1 contains the data collected for the elaboration of this study.
(XLSX)
